# Treatment outcomes of periprosthetic joint infections of the elbow: a retrospective cohort study from a single referral centre

**DOI:** 10.1007/s00264-026-06736-z

**Published:** 2026-01-25

**Authors:** Matej Mazura, Michal Benes, David Veigl, Rastislav Hromadka, Stanislav Jr. Popelka, Vladislav Bartak

**Affiliations:** 1 1 st Department of Orthopaedics, First Faculty of Medicine, Charles University, Prague, Czech Republic; 2 Department of Anatomy, Second Faculty of Medicine, Charles University, Prague, Czech Republic

**Keywords:** Elbow arthroplasty, Periprosthetic joint infection, Revision surgery, DAIR, Two-stage exchange

## Abstract

**Purpose:**

Periprosthetic joint infection (PJI) is a potentially devastating complication following total elbow arthroplasty. In this retrospective study, we aimed to review our cohort of patients to assess the treatment success rates of elbow PJI using different treatment methods and to report on the additional treatment approach for failed cases.

**Methods:**

The study included 26 patients who underwent revision surgery for elbow PJI between 2007 and 2023. Patients were enrolled if they possessed a minimum follow-up of two years. Success of different treatment strategies, including debridement, antibiotics, and implant retention (DAIR) with or without modular component exchange, and two-stage revision was evaluated, as well as the fate of patients who experienced initial treatment failure.

**Results:**

At a mean follow-up of 140.5 ± 74.5 months, only 11 patients (42.3%) were successfully treated after a single revision. The overall infection-free survival rates were 65.4% at one year, 50.0% at two years, and 45.8% at five years. DAIR without component exchange had the lowest success (23.1%), while DAIR with modular component exchange and two-stage revision showed the highest (60.0% and 62.5%, respectively). Failed cases (57.7%) required a mean of 2.1 additional procedures to achieve infection control.

**Conclusion:**

Treatment of elbow PJI remains especially challenging due to an overall high treatment failure. While two-stage exchange appears to be the most effective treatment modality, DAIR with modular component exchange shows promising outcomes in well-fixed prostheses. DAIR without modular components exchange yields poor infection control rates and should be omitted.

## Introduction

Total elbow arthroplasty (TEA) remains an increasingly utilized reconstructive procedure, with the most common indication for implantation being rheumatoid destruction. The development of semi-constrained prostheses has expanded the indications to include conditions such as habitual elbow instability, as well as acute comminuted distal humerus fractures in the elderly [1]. Periprosthetic joint infection (PJI) represents a severe complication following TEA that can substantially impair the quality of life or even further compromise the host. According to the literature, the incidence of infectious complications after TEA ranges from 2.5% to 12% [2–5].

The diagnostic algorithms used for hip and knee PJI are not directly applicable to TEA, owing to differences in joint anatomy and clinical presentations. Laboratory markers have limited utility in diagnosing elbow PJI, as inflammatory markers may be elevated due to the underlying rheumatologic conditions [6, 7]. Currently, the most reliable diagnostic methods remain collection of microbiological cultures and intraoperative histological analysis; however, both are susceptible to sampling error [8, 9]. Surgical management options include debridement, antibiotics, and implant retention (DAIR) with or without modular component exchange; one-stage exchange or two-stage revision with spacer implantation; and simple prosthesis extraction. Although recent consensus recommendations for the evaluation and treatment of elbow PJI have been published based on the 2018 International Consensus Meeting, elbow PJI treatment decisions have historically been guided primarily by clinical judgement and extrapolated from the hip and knee PJI literature [10, 11]. Thus, expanding the scientific literature reporting on treatment experience with elbow PJI is essential in order to implement appropriate treatment strategies. To date, studies have generally included only small patient cohorts [8, 12], and long-term outcomes are limited, despite their high value for proper PJI management.

This study aimed to evaluate our cohort of patients who underwent surgical revision for PJI of the TEA at a single referral centre in order to evaluate the treatment success rate of different methods, and to assess the fate of patients who experienced initial treatment failure.

## Materials and methods

After obtaining the institutional review board approval, our institutional database was retrospectively searched to identify all patients treated for PJI of the elbow from January 2007. All patients were managed at a single referral musculoskeletal infection center by members of the specialized team. Owing to the referral nature of our center, the overall incidence of elbow PJI could not be established since the institution handles infectious complications from the whole country.

### Inclusion and exclusion criteria

**Fig. 1:**
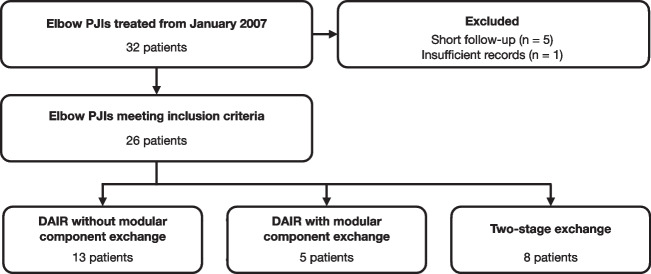
Flowchart showing the process of patient selection

Patients were included if they had a minimum follow-up of two years since the initial treatment for PJI, and possessed complete medical records. Exclusion criteria met patients who had negative intraoperative cultures or PCR together with inconclusive clinical findings, who were lost to follow-up, and had insufficient medical records. After applying the aforementioned criteria, 26 patients were deemed eligible for inclusion in the study (Fig. 1).

### Cohort characteristics

Out of the 26 eligible patients, there were 17 females (65.4%) and nine males (34.6%) with a mean age of 61.0 ± 10.1 (range 40–77) years. Rheumatic destruction of the elbow was the most common indication for TEA (23 cases; 88.5%). All of these patients with rheumatic disease received immunosuppressants prior to the onset of the PJI. PJI developed at a mean of 67.4 ± 79.9 months since the index procedure. *Staphylococcus aureus* was the most commonly isolated organism (15 cases; 57.7%), including three methicillin-resistant cases, followed by *Staphylococcus epidermidis* (five cases; 19.2%), *Streptococcus agalactiae* (one case; 3.8%), *Staphylococcus hominis* (one case; 3.8%), *Streptococcus mitis* (one case; 3.8%), and three cases (11.5%) possessed mixed organisms. For comparative analysis, we considered *Staphylococcus aureus*, *Enterococcus* species, beta-hemolytic *Streptococci*, gram-negative rods, and fungi as high-virulence organisms. Complete baseline characteristics of the cohort are summarized in Table 1.

**Table 1:** Baseline characteristics of the cohort

Age	61.0 ± 10.1
Female sex	17 (65.4%)
Charlson Comorbidity Index	4.1 ± 1.8
Diabetes	3 (11.5%)
Oncological disease	0 (0%)
Number of previous surgeries	0.3 ± 0.7
Follow-up (months)	140.5 ± 74.5
Indication for TEA	
Rheumatoid arthritis	23 (88.5%)
Post-traumatic	3 (11.5%)
Type of prosthesis	
Acclaim	3 (11.5%)
Coonrad-Morrey	6 (23.1%)
Discovery	15 (57.7%)
Custom-made	1 (3.8%)
Unknown	1 (3.8%)

### Surgical technique and treatment protocol

All patients with suspected PJI of the elbow were evaluated according to the diagnostic criteria of the Musculoskeletal Infection Society (MSIS, 2018). The treatment strategy was individualized based on the duration of symptoms, implant stability, bone stock, soft-tissue condition, and overall patient health status. The decision on the method relied on the treating physician and patients status. Generally, we attempt to adhere to the following protocols. In cases of early postoperative or acute haematogenous infection (< four weeks following implantation or symptom onset), a DAIR procedure was performed. During the procedure, multiple intraoperative samples (at least four) were obtained for microbiological culture and PCR analysis. This was followed by meticulous radical debridement of all necrotic and infected tissues and extensive synovectomy. Intraoperative lavage was carried out using at least nine litres of sterile saline solution combined with a local antiseptic agent. The polyethylene insert and other modular components were exchanged whenever technically feasible. Local antibiotic carrier was applied into the joint cavity based on the surgeons preference. Whenever possible, the antibiotic selection was tailored to the sensitivity of the identified pathogen. For chronic infections (> four weeks since the index procedure), a two-stage revision procedure was the treatment of choice. During the first stage, all prosthetic components and as much cement as possible were removed. Extensive debridement was then performed. Depending on the intraoperative findings and soft-tissue conditions, either an antibiotic-loaded cement spacer was implanted, or in selected cases, only prosthesis extraction without spacer placement was performed. After an eight week course of targeted antibiotic therapy and confirmation of infection eradication (decrease in CRP, ESR, and negative aspiration culture), second-stage reimplantation was carried out. One-stage exchange was not performed as a primary treatment in any of the finally included patients.

Antibiotic regimens were selected in close cooperation with infectious disease specialists, microbiologists, and clinical pharmacists. The total duration of antibiotic therapy was twelve weeks for DAIR procedures, including at least two weeks of intravenous administration, followed by oral antibiotics according to pathogen sensitivity and clinical response. For two-stage revisions, the duration of systemic antibiotic therapy was six to eight weeks.

In case of no complications, patients were clinically and radiographically followed at six weeks, three months, six months, and annually thereafter.

### Outcome variables

Patient records were manually reviewed to compile data on patient status based on the Charlson comorbidity index and other comorbidities, indication for TEA, number of previous procedures, used implant, organism information, type of revision procedure for PJI, presence of a sinus tract, use of antibiotic-loaded carrier, and mortality. Treatment failure was considered when the same pathogen was identified in the postoperative period (considered as relapse) or a new pathogen occurred in the subsequent microbiological testing (considered as reinfection). Primary outcome was to state the overall success rate of the initial treatment. Secondary outcomes were to analyze difference in baseline characteristics between patients who were successfully treated and patients who failed initial treatment. Additionally, we aimed to assess the fate of the initially failed cases.

### Statistical analysis

Basic descriptive statistics were used for presentation of categorical and continuous data. Categorical variables were compared using Fisher exact test while continuous variables were analyzed using Mann–Whitney *U* test. Kaplan–Meier survival curves were constructed to evaluate the survivorship of different treatment methods with revision procedure or infection recurrence as endpoints. Differences in survivorship were compared using the log-rank test. Level of statistical significance was set at *P* < 0.05.

## Results

During the mean 140.5 ± 74.5 months follow-up period, 11 patients (42.3%) were successfully treated with a single revision procedure, while the initial treatment failed in 15 patients (57.7%). DAIR was the most commonly performed procedure (18 cases; 69.2%), including 13 cases (72.2%) without modular component exchange and five cases (27.8%) with modular component exchange. The remaining eight cases (30.8%) were treated with two-stage exchange.

**Fig. 2:**
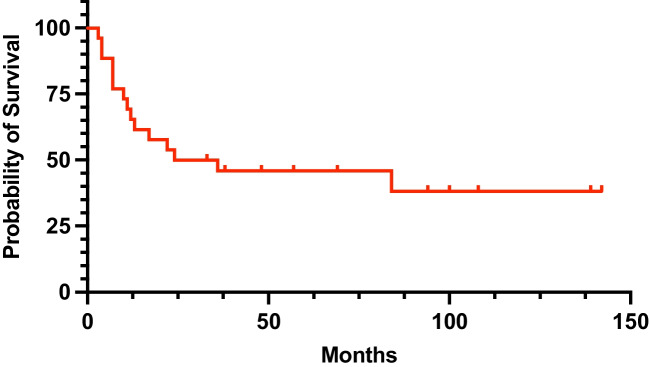
Kaplan–Meier curve showing the overall infection-free survival following the initial treatment

**Fig. 3:**
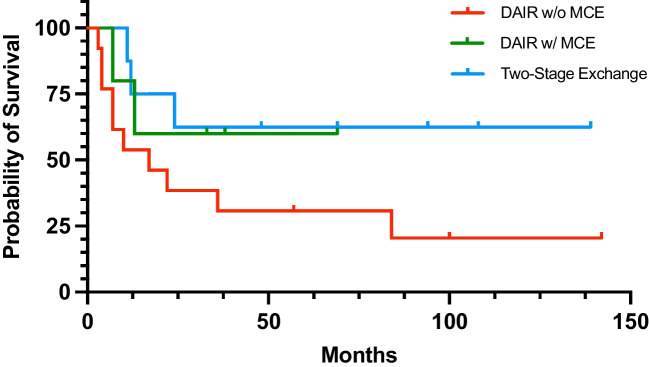
Kaplan–Meier curve showing the infection-free survival of different treatment methods

The overall infection-free survival following the initial treatment was 65.4% at one year, 50.0% at two years, and 45.8% at five years (Fig. 2). DAIR without modular component exchange demonstrated the lowest success rate of 23.1%. Treatment success of DAIR was higher when modular components were exchanged (60.0%). Comparable, yet slightly improved, success rate was identified in patients treated with two-stage exchange (62.5%). When stratified by initial PJI treatment, infection-free survival at two years was 38.5% for patients who underwent DAIR without modular component exchange, 60.0% for patients undergoing DAIR with modular component exchange, and 75.0% for patients treated with two-stage exchange (Fig. 3). However, given the small sample size, statistically significant difference in survivorship was not reached (*P* = 0.165) (Fig. 3).

Comparative analysis showed that there were no statistically significant differences in the baseline characteristics between the successfully treated patients and the patients who experienced initial treatment failure, including the rates of infection with high-virulence organisms (Table 2).

**Table 2:** Comparison of variables in treatment success group versus treatment failure group following the initial treatment

Variable	Success (*n* = 11)	Failure (*n* = 15)	*P*
Age	59.5 ± 11.1	62.1 ± 9.5	0.549
Female sex	7 (63.6%)	10 (66.7%)	0.999
Charlson Comorbidity Index	3.5 ± 1.8	4.5 ± 1.8	0.205
Number of previous surgeries	0.5 ± 1.0	0.1 ± 0.4	0.326
Indication for TEA			
Rheumatoid arthritis	9 (81.8%)	14 (93.3%)	0.556
Post-traumatic	2 (18.2%)	1 (6.7%)	
Presence of sinus tract	8 (72.7%)	10 (66.7%)	0.999
Antibiotic-loaded carrier	5 (45.5%)	2 (13.3%)	0.178
High-virulence organism	9 (81.8%)	9 (60.0%)	0.395

Fifteen patients (57.7%) experienced initial treatment failure due to infection relapse (eight cases; 53.3%) or reinfection (seven cases; 46.7%), and required a mean of 2.1 ± 1.6 additional procedures (Table 3). Repeated DAIR was performed in ten cases, however, only in five cases DAIR was also the final treatment. Importantly, a single DAIR was not efficient in any of the failed cases, with all successfully treated patients by DAIR requiring at least two additional procedures. Seven patients (46.7%) retained their fixed implants at the final follow-up, while eight patients (53.3%) required implant extraction with or without subsequent reimplantation. The mean time to revision after the first PJI treatment was 17.4 ± 20.5 months. As a definitive treatment, six patients (40.0%) underwent DAIR; five patients (33.3%) underwent permanent resection arthroplasty; two patients (13.3%) were treated with two-stage exchange; one patient (6.7%) underwent one-stage exchange; and one patient (6.7%) received chronic antibiotic suppression (Table 3).

**Table 3:** Management of patients who experienced failure of the initial treatment

Case	Sex	Age	Organism	Type of initial treatment	Recurrence type	Additional treatment	Final treatment
1	F	70	MRSA	DAIR w/o MCE	relapse	DAIR w/o MCE (4x)	Resection arthroplasty
2	F	70	mixed	DAIR w/o MCE	reinfection	DAIR w/o MCE (5x)	DAIR w/o MCE
3	F	77	mixed	DAIR w/o MCE	relapse	DAIR w/o MCE (1x)	DAIR w/o MCE
4	M	47	MSSA	DAIR w/o MCE	relapse	DAIR w/o MCE (1x)	Resection arthroplasty
5	F	49	*Streptococcus agalactiae*	DAIR w/o MCE	relapse	DAIR w/o MCE (1x)	DAIR w/o MCE
6	M	57	MSSA	DAIR w/o MCE	reinfection	DAIR w/o MCE (1x)	DAIR w/MCE
7	F	69	MRSA	DAIR w/o MCE	relapse	-	Two-stage exchange
8	F	73	*Staphylococcus epidermidis*	DAIR w/o MCE	relapse	DAIR w/o MCE (1x)	DAIR w/o MCE
9	M	58	*Staphylococcus hominis*	DAIR w/o MCE	reinfection	DAIR w/o MCE (1x)	Resection arthroplasty
10	M	52	MSSA	DAIR w/o MCE	relapse	-	Resection arthroplasty
11	F	54	MSSA	DAIR w/MCE	relapse	DAIR w/MCE (1x)	Resection arthroplasty
12	F	63	*Streptococcus mitis*	DAIR w/MCE	reinfection	-	Two-stage exchange
13	F	64	*Staphylococcus epidermidis*	DAIR w/o MCE	reinfection	DAIR w/o MCE (2x)	One-stage exchange
14	F	63	*Staphylococcus epidermidis*	Two-stage exchange	reinfection	-	Chronic ATB suppression
15	M	73	MSSA	Two-stage exchange	reinfection	-	DAIR w/o MCE

## Discussion

While there is a greater understanding of the management of hip and knee PJI, there is a gap in the current literature regarding the optimal management of elbow PJI, [13] albeit the infectious complications of TEA has proved to be difficult-to-treat and are responsible for significant patient morbidity. This retrospective cohort study evaluated outcomes after surgical revision for elbow PJI in a single high‑volume musculoskeletal infection center. With our contribution we aim to report on the success rates of different treatment strategies utilized in septic TEA revisions. Over an 18 years of our experience, more than half of the initial treatments failed. In light of the different treatment modalities used in our cohort of patients, we highlight the recognition of the obsolete role of DAIR without modular component exchange which by far yielded the worst outcomes.

The most commonly employed treatment method in our cohort was DAIR, particularly without modular component exchange. However, this strategy demonstrated the poorest success rate (23.1%), Conversely, DAIR with modular component exchange demonstrated a notably improved success rate (60.0%). The markedly better outcomes when modular components were exchanged during DAIR compared with DAIR without exchange emphasize the importance of addressing potential biofilm-laden during initial revision whenever feasible. This trend is consistent with the broader PJI literature, where modular component exchange during DAIR is strongly advisable since it significantly improves the outcomes [14].

Although our data suggested a trend toward better infection-free survival in the two-stage revision group, the difference did not reach statistical significance, likely due to the small sample size. Nonetheless, the survival analysis revealed a clear hierarchy in outcomes, with two-stage exchange outperforming both DAIR strategies at two year follow-up (75.0% vs. 60.0% vs. 38.5%, respectively). Two-stage revision showed the highest success rate at 62.5%, in line with the hip and knee arthroplasty literature, where this approach is considered the gold standard for chronic PJI [15].

Considerable proportion of treatment failures occurred despite the absence of significant differences in baseline characteristics between successful and unsuccessful cases. A substantial proportion of patients who failed initial treatment required multiple procedures and ultimately implant extraction with or without subsequent reimplantation. Interestingly, no patient who was successfully treated with DAIR as a final treatment did so after only one procedure, reinforcing the notion that single-stage DAIR may be insufficient in many cases. Although prompt DAIR was performed in all cases, we could not obtain exact timing from the onset of symptoms to surgery for additional analysis, which is known to impact the treatment success. Therefore, we can only theorize on the efficacy of repeated DAIR. Moreover, the high rate of implant retention (46.7%) in failed cases at final follow-up reflects the limitations imposed by poor bone stock, host factors, or technical constraints that often preclude safe prosthesis exchange [16].

Currently, insufficient studies are addressing the clinical outcomes of DAIR in elbow TEA. The largest study by Tai et al. includes 26 elbows and reports a 65% failure rate within two years [12]. They reported a median time to failure of 43 days [12]. In a systematic review, the overall success rate was calculated to be 55.8% (range: 11.1%−80.9%) [8]. In our study, the success rate of DAIR reached 60%when modular components were exhanged. These results are consistent with the available literature.

Performing DAIR for TEA infections should be considered with careful attention to the principles established for PJI treatment in the hip and knee, such as timing of the intervention, knowledge of a sensitive pathogen, patient’s systemic status, and any ongoing immunosuppressive therapy. Based on both existing literature and our own experience, two-stage exchange with an antibiotic-loaded cement spacer remains the gold standard for managing elbow PJI. It is important to emphasize that successful two-stage exchange requires complete removal of the original endoprosthesis, including an attempt to extract all bone cement from the medullary canals. This step is often technically challenging, and due to associated bone loss, it may be more advantageous to leave the cement in situ. As such, treatment decisions must always take into account patient comorbidities, surgical risks, and individual expectations, ensuring that the therapeutic goal is clearly defined and realistically achievable.

Several limitations of this study must be acknowledged. The retrospective design introduces potential selection and information bias. Another limitation of this study is the missing timeframe from the onset of symptoms to the treatment, potentially impacting the success rate of different surgical techniques. Outcomes such as functional status and patient-reported outcomes were not systematically collected, which restricts the assessment of quality of life following the elbow PJI treatment. With each revision procedure, we logically suspect that the elbow functionality decreases but we were not able to compare functional outcomes among the different treatment methods. Moreover, the treatment strategies evolved during the study period, including innovations in implant designs which allowed, for example, for routine exchange of modular components. This fact may lead to differences in treatment approaches over time. Additionally, since the study was performed at a tertiary referral center, more complex cases tend to be referred to our institution if they cannot be handled in the primary medical facility. Although this study poses several limitations, it provides evidence on various surgical strategies used for management of elbow PJI, and may serve as valuable information source for more robust pooled analysis.

## Conclusions

Periprosthetic joint infection following total elbow arthroplasty remains a highly challenging complication, associated with substantial patient morbidity and frequent treatment failure. The overall success rate of initial surgical intervention was modest, with more than half of the patients requiring additional procedures. DAIR without modular component exchange led to a very poor success rate (23.1%), thus it should be limited only for very frail patients due to its inherently high failure rates, and modular components should be exchanged whenever possible. DAIR with modular component exchange and two-stage exchange achieved nearly similar overall success rates (60.0% and 62.5%, respectively). Nonetheless, the high rate of subsequent procedures and the substantial proportion of patients requiring implant removal emphasize the need to define optimal treatment strategy and establish standardized protocols.

## Data Availability

No datasets were generated or analysed during the current study.
